# Thermoelectric Characteristics of Self-Supporting WSe_2_-Nanosheet/PEDOT-Nanowire Composite Films

**DOI:** 10.1021/acsami.3c02660

**Published:** 2023-07-14

**Authors:** Sisi Guo, Qiufeng Meng, Jie Qin, Yong Du, Lei Wang, Per Eklund, Arnaud le Febvrier

**Affiliations:** † School of Materials Science and Engineering, Shanghai Institute of Technology, 100 Haiquan Road, Shanghai 201418, China; ‡ Thin Film Physics Division, Department of Physics, Chemistry and Biology (IFM), Linköping University, E-58183 Linköping, Sweden

**Keywords:** thermoelectric, composite, WSe_2_, conducting polymer, flexibility

## Abstract

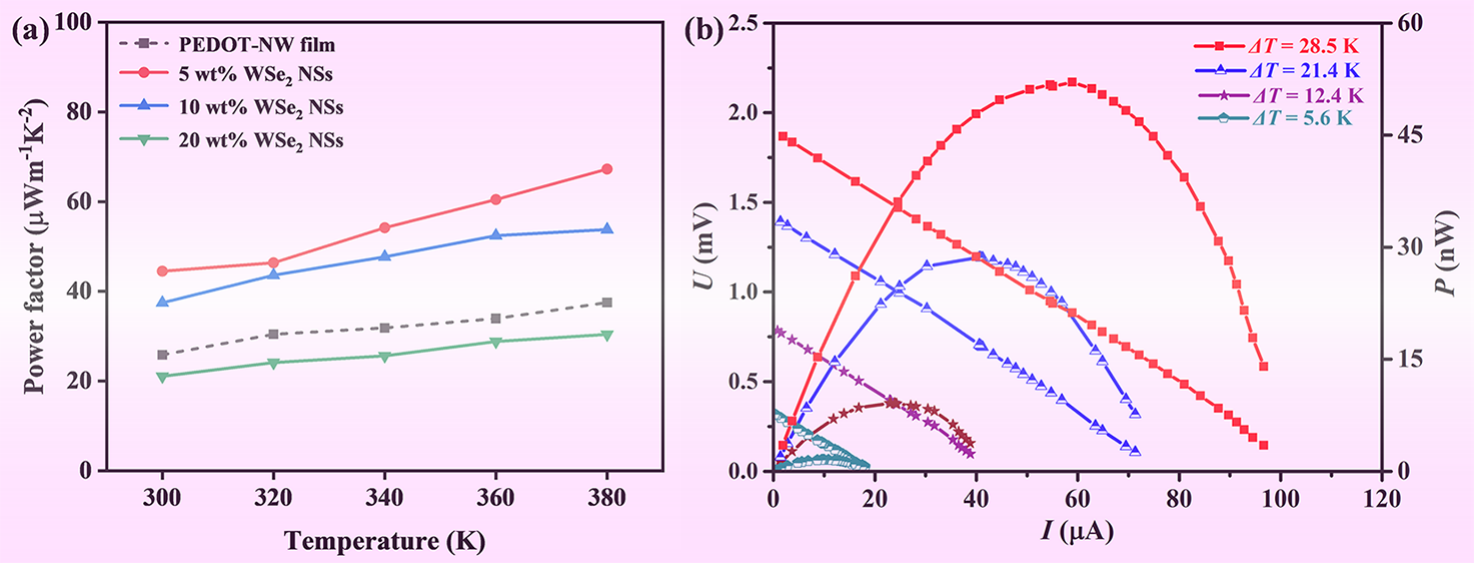

Conducting polymer poly(3,4-ethylenedioxythiophene) nanowires (PEDOT NWs) were synthesized by a modified self-assembled micellar soft-template method, followed by fabrication by vacuum filtration of self-supporting exfoliated WSe_2_-nanosheet (NS)/PEDOT-NW composite films. The results showed that as the mass fractions of WSe_2_ NSs increased from 0 to 20 wt % in the composite films, the electrical conductivity of the samples decreased from ∼1700 to ∼400 S cm^–1^, and the Seebeck coefficient increased from 12.3 to 23.1 μV K^–1^ at 300 K. A room-temperature power factor of 44.5 μW m^–1^ K^–2^ was achieved at 300 K for the sample containing 5 wt % WSe_2_ NSs, and a power factor of 67.3 μW m^–1^ K^–2^ was obtained at 380 K. The composite film containing 5 wt % WSe_2_ NSs was mechanically flexible, as shown by its resistance change ratio of 7.1% after bending for 500 cycles at a bending radius of 4 mm. A flexible thermoelectric (TE) power generator containing four TE legs could generate an output power of 52.1 nW at a temperature difference of 28.5 K, corresponding to a power density of ∼0.33 W/m^2^. This work demonstrates that the fabrication of inorganic nanosheet/organic nanowire TE composites is an approach to improve the TE properties of conducting polymers.

## 1 Introduction

Thermoelectric (TE) materials can directly convert thermal and electrical energies.^1,2^ TE generators can provide electrical energy for wearable and portable electronic equipment using low-level-grade heat energy and have the advantages of no moving parts, long service life, and no noise.^3,4^ The efficiency of TE generators^5^ is related to the dimensionless figure of merit *ZT* (*ZT* = *S*^2^σ*T*/κ), where σ, *S*, κ, and *T* are the electrical conductivity (S cm^–1^), Seebeck coefficient (μV K^–1^), thermal conductivity (W m^–1^ K^–1^), and temperature in Kelvin (K), respectively. Mechanically flexible TE materials are useful in wearable applications and for improved connection to heat sources.^6−9^ Conventionally, conducting polymer-based materials and devices are used for this purpose.^10−12^

Poly(3,4-ethylenedioxythiophene) (PEDOT) is important for polymeric TE materials because of its high electrical conductivity^12−14^ and adjustable morphology that can be tuned by adjusting the synthesis conditions. While PEDOT in itself has modest thermoelectric properties, PEDOT nanofibers can exhibit higher thermoelectric properties. Hu et al.^15^ synthesized PEDOT with different nanostructures through chemical oxidation polymerization and found that one-dimensional PEDOT nanofibers yielded a high power factor (*S*^2^σ) of 16.4 μW m^–1^ K^–2^. Therefore, preparation of one-dimensional PEDOT structures may be of significance to improve the TE properties of PEDOT-based materials. To this end, the self-assembled micellar soft-template method is easy to operate and suitable for large-scale production, and many researchers investigated the synthesis of one-dimensional PEDOT structures in this method.^14,16,17^

Beyond PEDOT-based nanostructures, introducing thin or two-dimensional inorganic materials in inorganic/organic composites offers further possibilities for improved TE materials and devices. Transition-metal dichalcogenides (TMDCs), whose chemical formula can be expressed as MX_2_, in particular, sulfides and selenides, exhibit good thermoelectric properties.^18−20^ Mixing TMDCs with conducting polymers to prepare organic/inorganic composites is thus an approach to enhance the TE properties of conducting polymers. Jiang et al.^21^ synthesized molybdenum disulfide (MoS_2_) nanosheets (NSs) by a liquid exfoliation method and then prepared poly(3,4-ethylenedioxythiophene):poly(4-styrenesulfonate) (PEDOT:PSS)/MoS_2_ composite films by the direct vacuum filtration method, yielding a room-temperature power factor of 45.6 μW m^–1^ K^–2^ for a sample with 4 wt % MoS_2_ NSs. MoSe_2_/PEDOT:PSS^22^ and WS_2_/PEDOT:PSS^23^ composite films were also prepared by the same process and exhibit similar power factor values.

Tungsten diselenide (WSe_2_) is a semiconducting TMDC with a band gap of ∼1.5 eV,^24^ high mobility,^25^ and good thermoelectric properties.^20,26^ This suggests that WSe_2_-NS/PEDOT-NW composites would be of interest for flexible thermoelectrics, but such nanocomposites have not yet been reported. Here, we synthesized one-dimensional PEDOT NWs through a modified self-assembled micellar soft-template method and prepared two-dimensional WSe_2_ NSs by lithium ion (Li^+^) intercalation––hydrothermal method. Flexible and self-supporting WSe_2_-NS/PEDOT-NW composite films were prepared by a vacuum filtration method. The effects of the contents of WSe_2_ NSs on microstructures, compositions, TE performance, as well as mechanical flexibility of the composite films have been investigated.

## 2 Experimental Section

### 2.1 Raw Materials

EDOT monomer (chemical grade), lithium hydroxide monohydrate (LiOH·H_2_O, 99%, guaranteed reagent), anhydrous ferric chloride (FeCl_3_, ≥99%), sodium dodecyl sulfate (SDS, pharmaceutical grade), ethylene glycol (EG, ≥99.5%, analytical reagent), acetone (≥99.5%, analytical reagent), methanol (≥99.5%, analytical reagent), and nylon membrane (the average pore size was 0.22 μm) were bought from Shanghai Titan Scientific Co., Ltd., China. WSe_2_ (>99%) was bought from Nanjing JCNANO Technology Co., Ltd., China. All chemicals were used as received without further purification.

### 2.2 Synthesis of PEDOT NWs

PEDOT NWs were synthesized by a modified self-assembled micellar soft-template method. 10 mmol SDS powder was dissolved in 100 mL of deionized water under stirring to form a homogeneous SDS solution. 15 mmol FeCl_3_ was added into the solution and stirred for 1.5 h at 50 °C, and the color of the mixture solution changed into dark yellow. Afterwards, 7 mmol EDOT monomers were added into the above mixture solution and polymerized at 50 °C for 6 h. After the reaction, the precipitates were left to cool down to room temperature in an oil bath. Then, the PEDOT-NW precipitates were centrifuged at 6500 rpm for 5 min and washed with methanol until the supernatant was colorless. The synthesized PEDOT NWs were dispersed in 500 mL of methanol with sonication to form a homogeneous dispersion.

### 2.3 Preparation of WSe_2_ NSs

WSe_2_ powders (0.5 g) and LiOH·H_2_O (0.48 g) were added into EG (60 mL) under stirring for 30 min. The mixture suspension was transferred to a Teflon-lined autoclave (100 mL) and reacted at 200 °C for 24 h to intercalate Li^+^ into WSe_2_ multilayers. After the reaction, the mixture suspension was naturally cooled down to room temperature and centrifuged at 10,000 rpm for 5 min, and the Li^+^-intercalated WSe_2_ precipitates were washed several times with acetone to eliminate the residual reactants. The Li^+^-intercalated WSe_2_ products were added into 100 mL of deionized water and sonicated at 300 W for 6 h. The dispersions were centrifuged at 3000 rpm for 10 min, and the supernatant was collected and filtered on a nylon membrane. After drying at 80 °C for 12 h in a vacuum atmosphere, the exfoliated WSe_2_ NSs were acquired.

### 2.4 Preparation of Self-Supporting WSe_2_-NS/PEDOT-NW Composite Films

A certain weight of WSe_2_ NSs was dispersed in 15 mL of PEDOT NW dispersion under sonication for 0.5 h. Flexible WSe_2_-NS/PEDOT-NW composite films were prepared by a vacuum filtration method on a nylon membrane and dried for 12 h at 60 °C in a vacuum atmosphere, in which the contents of WSe_2_ NSs were 0, 5, 10, and 20 wt %, respectively. The dried WSe_2_-NS/PEDOT-NW composite films were easy to peel off from the nylon substrate.

### 2.5 Assembly of Flexible TE Generators

Four TE legs (20 mm × 5 mm) were prepared by cutting the WSe_2_-NS/PEDOT-NW sample and pasting on a polyimide film with a double-sided adhesive. The spacing between two legs was 5 mm. The TE legs were connected in series via silver pastes, and the silver pastes were dried in a vacuum at 60 °C overnight.

### 2.6 Characterization and Measurement

The morphologies of PEDOT NWs and WSe_2_ were characterized by transmission electron microscopy (TEM, FEI Tecnai 12, USA and FEI Talos F200x G2, USA). The microstructures and morphologies of the films were imaged by scanning electron microscopy (SEM, FEI Quanta 200 FEG, the Netherlands, and ZEISS Sigma 300, Germany). SEM mapping was performed with energy-dispersive spectroscopy (EDS, OXFORD Xplore 30, UK). X-ray photoelectron spectroscopy (XPS) and Raman spectroscopy measurements of PEDOT-NW films were performed with a Thermo Fisher Scientific ESCALAB 250Xi instrument (USA) with an Al Kα X-ray source (1486.6 eV) and a DXR laser Raman spectrometer (Thermo Fisher Scientific, USA) using DPSS laser at an excitation wavelength of 532 nm. The phase compositions of PEDOT NWs, WSe_2_, and WSe_2_-NS/PEDOT-NW samples were characterized by X-ray diffraction (XRD, D/max 2200PC, Japan) with Cu k_α_ radiation (λ = 1.54056 Å). The roughness of the PEDOT-NW film and WSe_2_-NS/PEDOT-NW composite film was obtained by using an atomic force microscope (Bruker Dimension Icon, Germany).

The Seebeck coefficient and electrical conductivity were measured in a low vacuum (≤40 Pa) from 300 to 380 K by a thin-film TE test system (MRS-3, Wuhan Giant Instrument Technology Co., Ltd., China). The flexibility of the PEDOT-NW film and WSe_2_-NS/PEDOT-NW composite film was studied by a home-made equipment at a bending radius of 4 mm, and the size of the tested sample was 20 mm in length and 5 mm in width.

The output performance of the TE generator was measured by a home-made equipment. During the measurement, the two ends of the tested generator along the length of the TE legs were placed on the Peltier platforms, and temperature differences were generated between the two ends of the generator by regulating the current generated by the source meter (Keithley 2400) passing through the Peltier platforms. The open-circuit voltage (*E*_oc_), output voltage (*U*) of TE generators were collected by a data acquisition instrument (Keysight 34970A). The output current (*I*) was calculated by dividing *U* by external resistive load (*R_EL_*). The output power (*P*) was measured by the following formula: *P* = *U* × *I*. The resistance change of the generator was tested by a home-made equipment when the generator was bent for different times at a radius of 15 mm.

## 3 Results and Discussion

**Figure 1:**
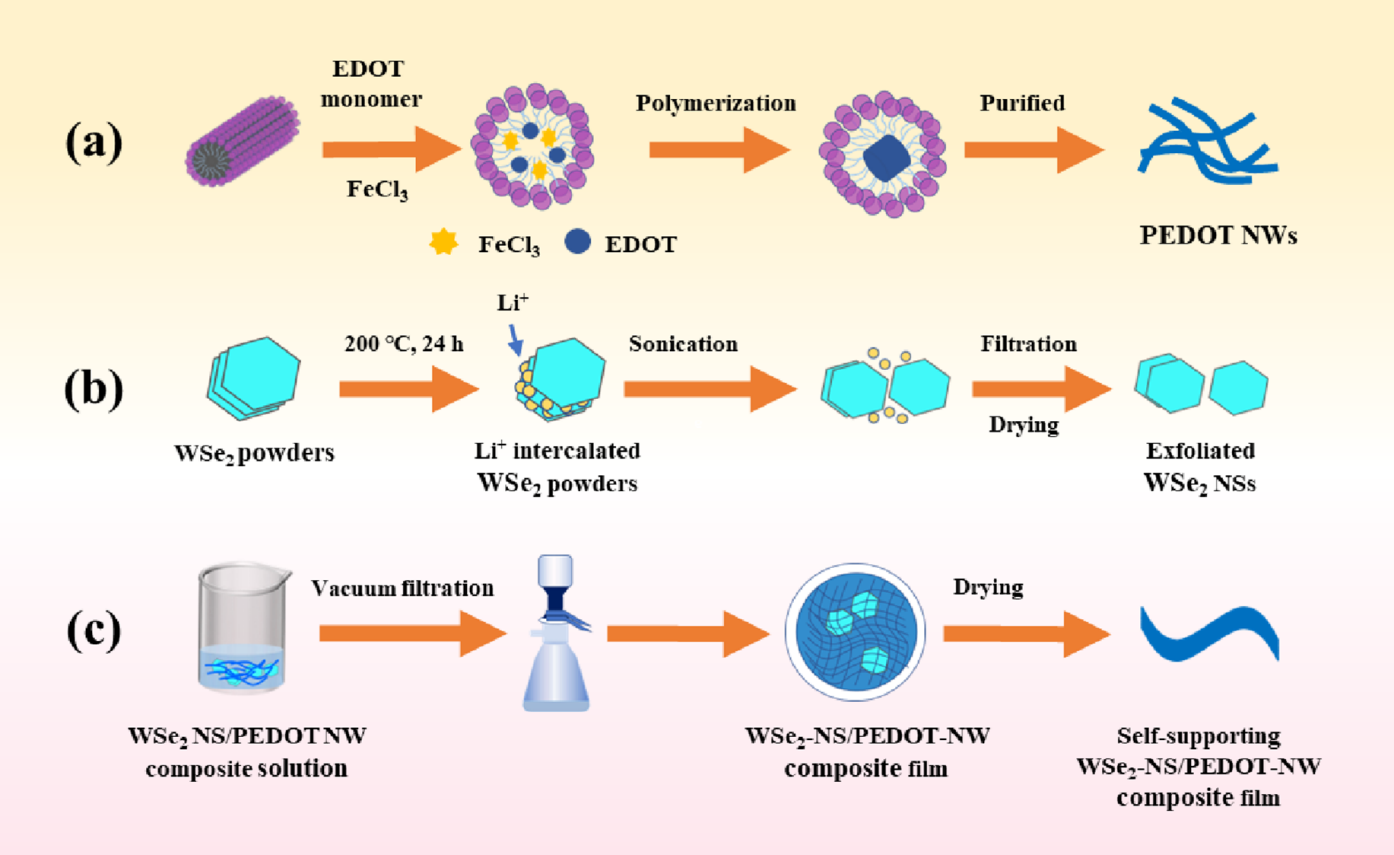
Schematic diagrams for the preparation procedures of (a) PEDOT NWs, (b) WSe_2_ NSs, and (c) freestanding WSe_2_-NS/PEDOT-NW composite film.

Figure 1a,b shows the schematic diagrams of the preparation procedures of PEDOT NWs and WSe_2_ NSs. Figure 1c shows a schematic diagram for the preparation procedure of flexible and self-supporting WSe_2_-NS/PEDOT-NW composite films.

**Figure 2:**
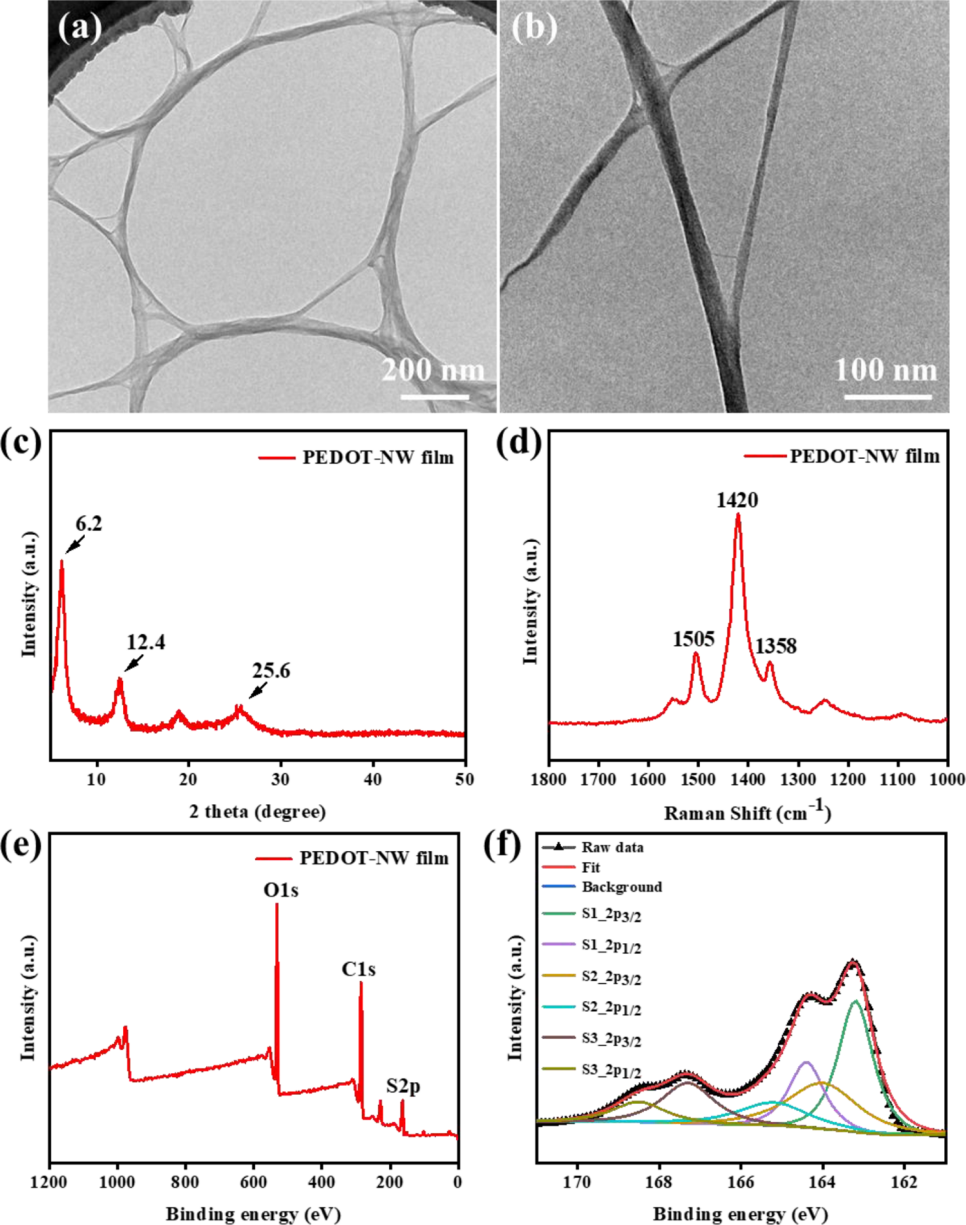
(a, b) TEM images of PEDOT NWs. (c) XRD patterns of the PEDOT-NW film. (d) Raman spectrum of the PEDOT-NW film. (e) Survey and (f) S2p XPS spectra of the PEDOT-NW film.

Figure 2a,b shows the TEM images of PEDOT NWs. The PEDOT NWs are entangled with each other into bundles due to their high surface energy as well as strong π–π stacking interactions between the PEDOT chains.^14,17^ The length and diameter of PEDOT NWs were measured (Figure 2a) by the ImageJ software. The length of PEDOT NWs was above 1000 nm, and the average diameter was ∼10 nm.

Figure 2c shows the XRD patterns of a PEDOT-NW film. The PEDOT NWs exhibit peaks at 2θ = 6.2°, 12.4°, 19.4°, and 25.6°, respectively, which can be assigned to the (100), (200), (400), and (020) crystal planes of PEDOT, respectively.^27−30^ The sharp peak at 2θ = 6.2° indicates satisfactory crystallinity of PEDOT NWs,^14^ and the peak at 2*θ* = 12.4° shows the degree of distortions from the ideal crystalline lattice in PEDOT NWs.^27^ The characteristic peak at 2*θ* = 25.6° is ascribed to the interchain planar ring-stacking distance.^16,27,30^ According to Bragg’s law (2d sin θ = *n*λ, where *d* represents the interplanar spacing, *θ* represents the angle between the incident X-ray and the relevant crystal plane, *n* is the diffraction order, and λ represents the X-ray wavelength),^31^ the π*–*π spacing distance of PEDOT NWs was 3.42 Å. Further insight into the composition of PEDOT NWs was provided by Raman (Figure 2d) and XPS (Figure 2e,f) analyses. As shown in Figure 2d, three bands can be observed at 1358, 1420, and 1505 cm^–1^, which correspond to C–C stretching, symmetric C=C stretching, and asymmetric C=C stretching, respectively.^32,33^ The elements C, O, and S were observed in the XPS survey spectrum of the PEDOT-NW film (Figure 2e). Figure 2f shows the S2p spectrum of the PEDOT-NW film; the doublets at 163.2 and 164.4 eV (S1_2p_3/2_ and S1_2p_1/2_) and 164.0 and 165.2 eV (S2_2p_3/2_ and S2_2p_1/2_) are ascribed to the neutral sulfur atoms (S^0^) and positively charged sulfur atoms (S^δ+^) from PEDOT chains.^14,16^ The higher binding energy doublets at 167.3 and 168.5 eV (S3_2p_3/2_ and S3_2p_1/2_) are attributed to the sulfur atoms of dodecyl sulfate anions (DS^–1^).^14^ These results show the synthesis of PEDOT NWs.

**Figure 3:**
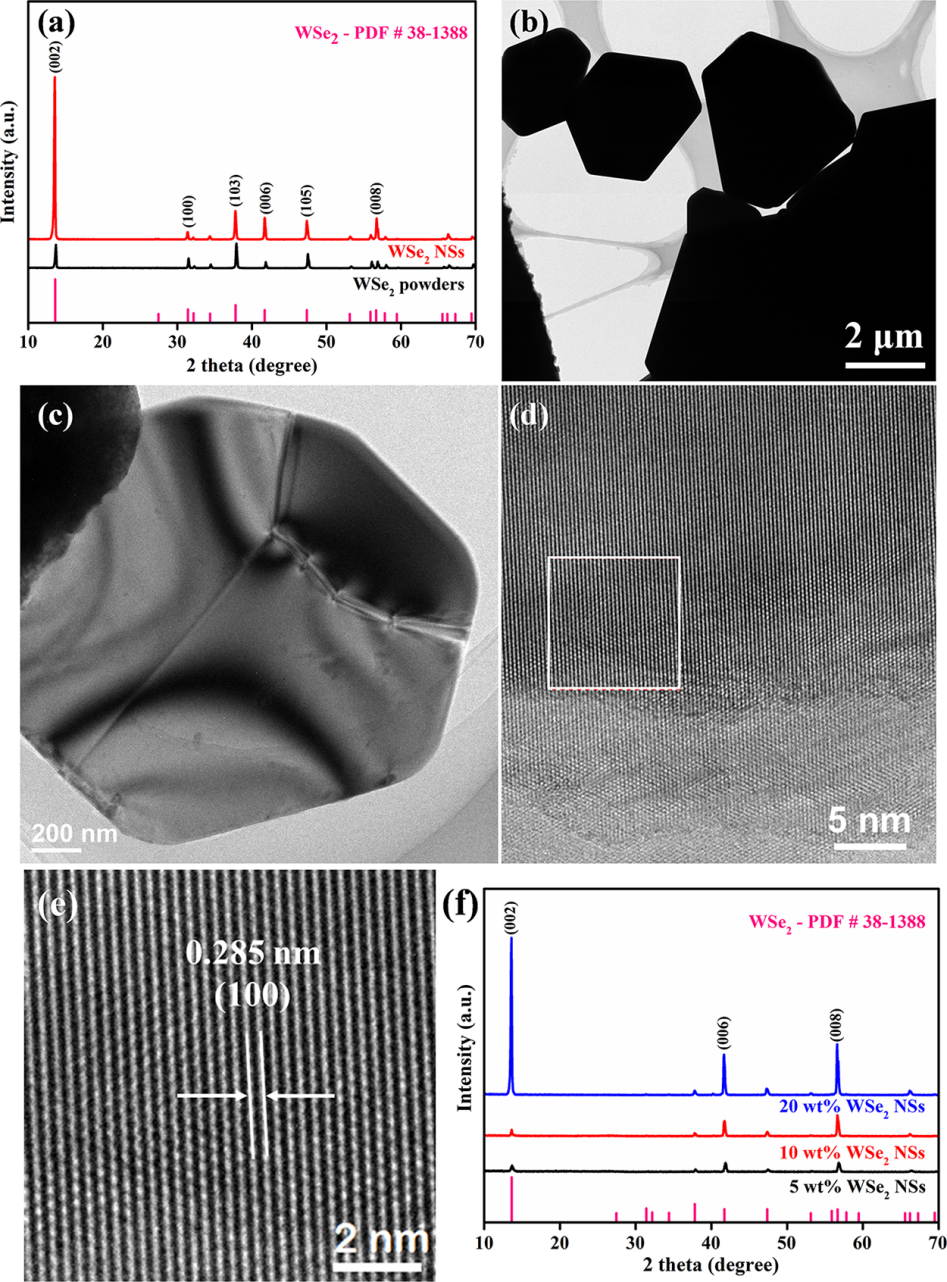
(a) XRD patterns of WSe_2_ and exfoliated WSe_2_ NS powders. TEM images of (b) WSe_2_ powders and (c, d) exfoliated WSe_2_ NSs. (e) HR-TEM image of WSe_2_ NSs. (f) XRD patterns of WSe_2_-NS/PEDOT-NW composite films with different mass fractions of WSe_2_ NSs.

Figure 3a shows the XRD patterns of WSe_2_ and exfoliated WSe_2_ NS powders. The characteristic peaks of WSe_2_ and exfoliated WSe_2_ NSs are consistent with the standard PDF card of WSe_2_ (no. 38-1388),^34^ and the relative peak intensities of WSe_2_ powders were changed after the exfoliation treatment. That is, the relative intensities at 2θ *=* 13.4°, 41.5°, and 56.5° corresponding to the (002), (006), and (008) planes of WSe_2_ were enhanced, while that at 2θ = 31.4° corresponding to the crystal plane of (100) was reduced, which infers that the exfoliated WSe_2_ NSs have a highly c-oriented crystal structure. This phenomenon is similar to a previous report of exfoliated MoSe_2_ NSs.^35^Figure 3b shows the TEM images of raw WSe_2_ powders; Figure 3c,d shows the TEM images of WSe_2_ NSs, revealing that the obtained nanosheets own a few layers. Figure 3e shows the high-resolution (HR-TEM) image. The lattice spacing is 0.285 nm, which correspond to the (100) crystal plane of WSe_2_ NSs. Figure 3f shows the XRD patterns of WSe_2_-NS/PEDOT-NW composite films. The peak intensities of the (002), (006), and (008) planes of WSe_2_ were enhanced as the content of WSe_2_ NSs increased in the composite film. The characteristic peaks of PEDOT NWs were not observed in the composite films, which might be because of the low intensities of PEDOT NWs.

**Figure 4:**
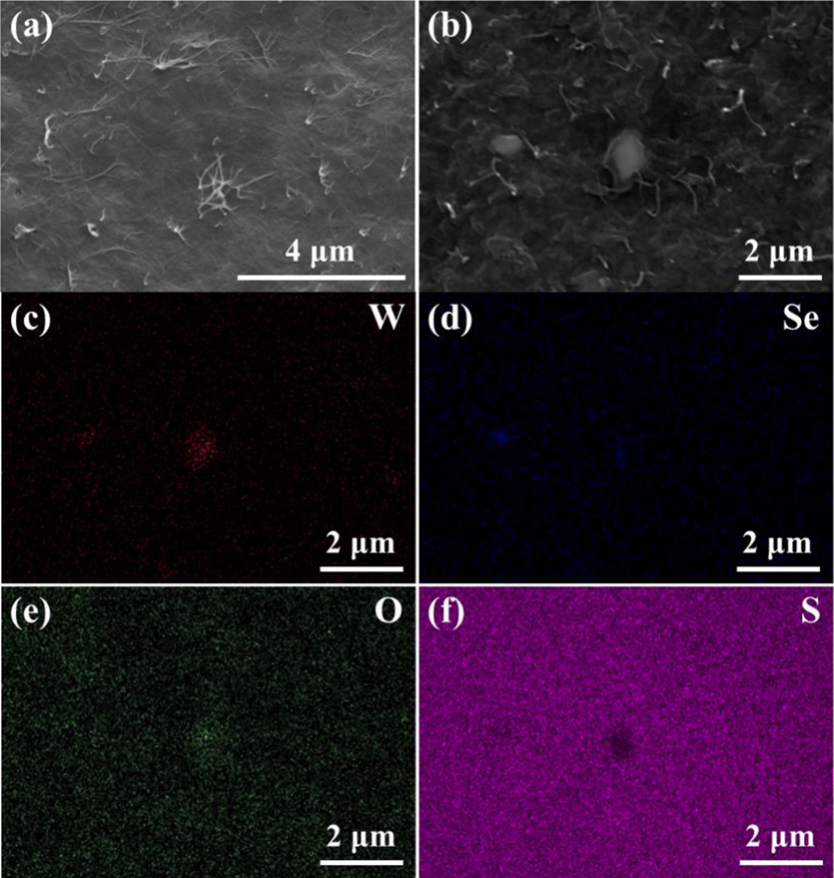
Surface SEM images of (a) PEDOT-NW film and (b) WSe_2_-NS/PEDOT-NW composite film with 5 wt % WSe_2_ NSs. SEM-EDS mappings of (c) W element, (c) Se element, (e) O element, and (f) S element corresponding to (b).

Figure 4a shows a surface SEM image of the PEDOT-NW film. The image shows that the PEDOT-NW film exhibits a relatively smooth morphology, and intersecting bundles of single PEDOT NWs can be clearly observed. Figure 4b,c–f shows the SEM image of a WSe_2_-NS/PEDOT-NW composite film with 5 wt % WSe_2_ NSs and the corresponding EDS mappings of W, Se, O, and S. The presence of W and Se is attributed to WSe_2_ NSs, while O and S come from PEDOT NWs. It is seen that WSe_2_ NSs are dispersed in the composite film, which demonstrates the synthesis of WSe_2_-NS/PEDOT-NW composite films. Moreover, after the addition of WSe_2_ NSs, the surface roughness of the composite film increased (see Figure 4a,b), and this phenomenon was also observed by the AFM analyses below.

**Figure 5:**
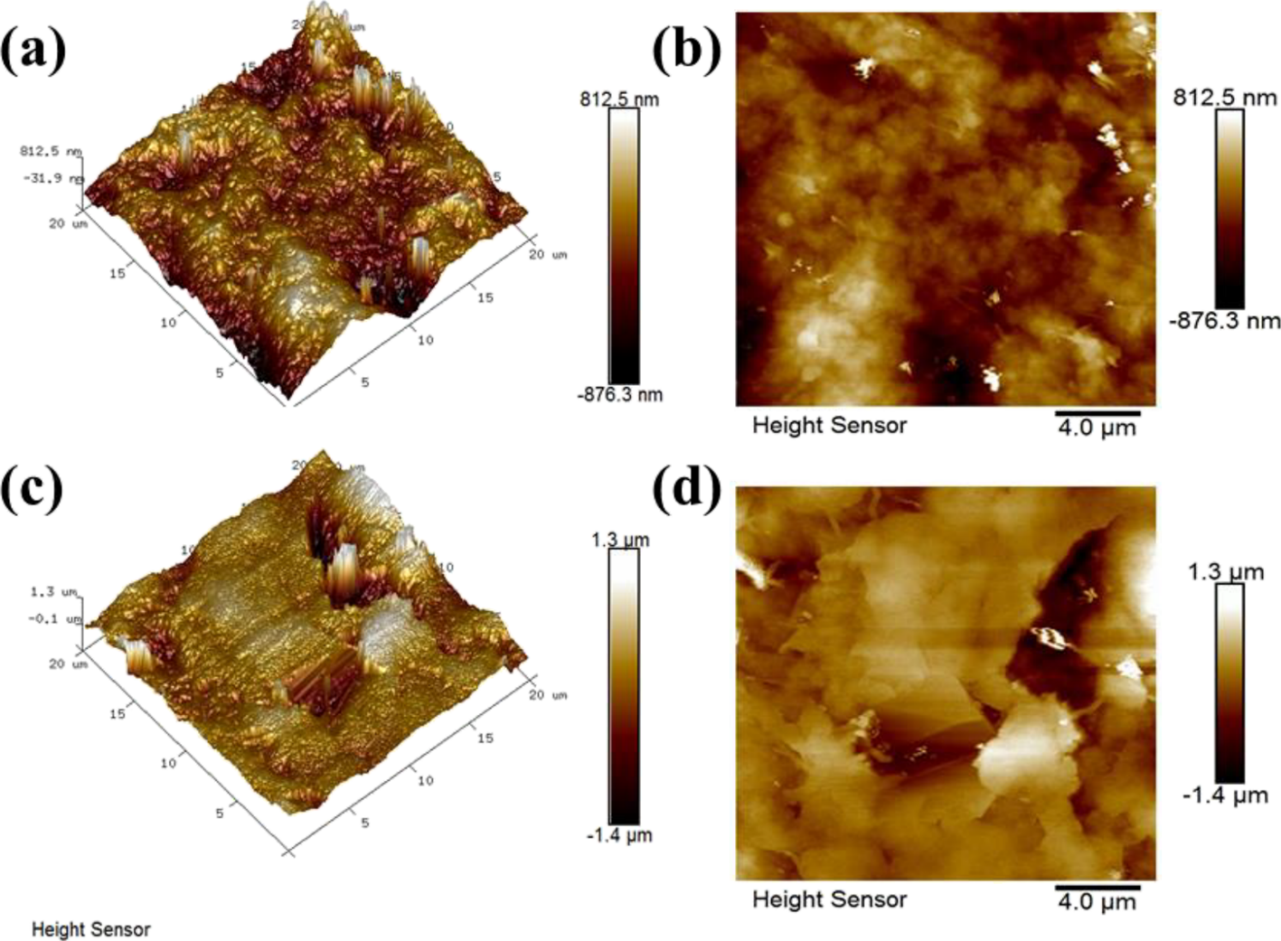
Surface topography measured by AFM. (a, b) Pristine PEDOT-NW film and (c, d) WSe_2_-NS/PEDOT-NW composite film with 5 wt % WSe_2_ NSs.

Figure 5 shows the surface topography of the PEDOT-NW film and WSe_2_-NS/PEDOT-NW composite film with 5 wt % WSe_2_ NSs. It can be seen that the surface root-mean-square roughness (*R*_rms_) values of PEDOT-NW and WSe_2_-NS/PEDOT-NW composite films were 233 and 324 nm, respectively.

**Figure 6:**
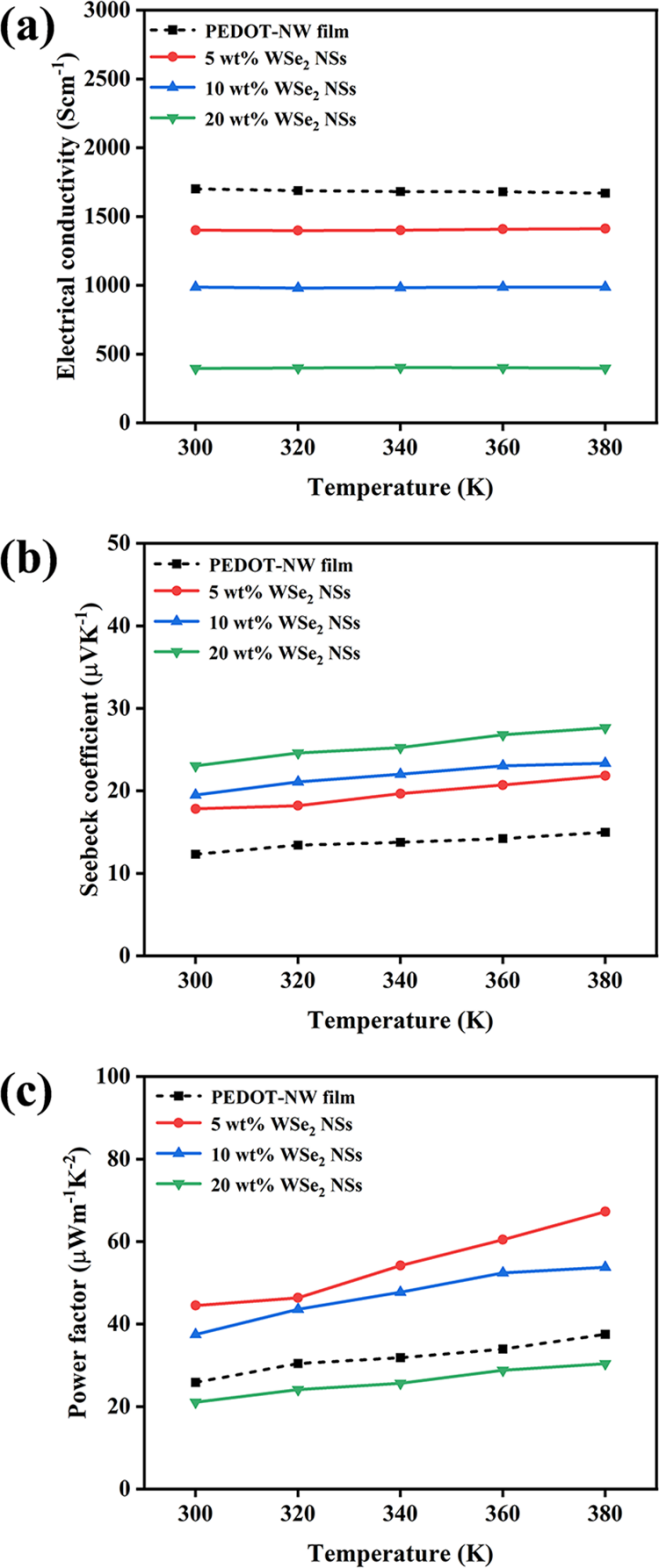
Temperature dependence of (a) σ, (b) *S*, and (c) power factor of WSe_2_-NS/PEDOT-NW composite films with different contents of WSe_2_ NSs.

Figure 6a displays the relationship between the temperature and electrical conductivity of WSe_2_-NS/PEDOT-NW composite films with different contents of WSe_2_ NSs. As the content of WSe_2_ NSs increased from 0 to 20 wt %, σ reduced from ∼1700 to ∼400 S cm^–1^ at 300 K. The reasons for the reduction of σ of the composite films may be due to the presence of fillers (WSe_2_ NSs) with different morphology and electrical behavior compared with the matrix (PEDOT NWs), which can alter the charge carrier concentration, carrier mobility, and thus the electrical conductivity. As the σ of WSe_2_ is very low (1.9 × 10^–3^ S cm^–1^ for WSe_2_ bulk at room temperature),^36^ the addition of WSe_2_ NSs would reduce the σ value of the WSe_2_-NS/PEDOT-NW composite films. The surface roughness of the composite (Figures 4 and 5) appears increased with the addition of WSe_2_ NSs, which may be detrimental to the carriers’ transportation.^37^ When the temperature rose from 300 to 380 K, σ of the WSe_2_-NS/PEDOT-NW composite films containing different mass fractions of WSe_2_ NSs was not significantly changed, and at 380 K, σ of the WSe_2_-NS/PEDOT-NW composite film with 5 wt % WSe_2_ NSs was 1412 S cm^–1^.

The carrier concentrations and mobilities were measured for different WSe_2_ content. With the increasing of WSe_2_ NS content, the carrier mobility is increased up to ∼85 cm^–2^ V^–1^ s^–1^, and the carrier concentration decreased to ∼5 × 10^19^ cm^–3^ for the sample with 20 wt% WSe2 NSs, resulting in the reduction of electrical conductivity and enhancement of the Seebeck coefficient.

Figure 6b presents the temperature dependence of *S* of WSe_2_-NS/PEDOT-NW composite films. *S* is positive, revealing that holes are the majority carriers.^38^*S* revealed an increasing tendency on the content of WSe_2_ NSs, and when the content of WSe_2_ NSs increased to 20 wt %, *S* increased to 23.1 μV K^–1^ at 300 K, which was ∼1.9 times than that of the PEDOT-NW film. WSe_2_ has high *S* (786.8 μV K^–1^ at room temperature for WSe_2_ bulk);^36^ thus the *S* value of the composite film could be enhanced after the addition of WSe_2_ NSs. Besides, the addition of WSe_2_ NSs can induce more interfaces between PEDOT NWs and WSe_2_ NSs, which may enhance the *S* value of the composite films by energy filtering.^39,40^ The *S* value of the composite films increased with the increasing temperature from 300 to 380 K, reaching a value of 27.7 μV K^–1^ for the sample containing 20 wt % WSe_2_ NSs. As a result, a power factor of 44.5 μW m^–1^ K^–2^ was achieved for the composite film with 5 wt % WSe_2_ NSs at 300 K (Figure 6c). As the temperature increased, a power factor of 67.3 μW m^–1^ K^–2^ was obtained at 380 K, which is higher than the previous reports on the MoS_2_/PEDOT:PSS composite film (45.6 μW m^–1^ K^–2^)^21^ and WS_2_/PEDOT:PSS composite film (45.2 μW m^–1^ K^–2^)^23^ but still lower than those of the 1-ethyl-3-methylimidazolium dicyanamide (EMIM:DCA)@PEDOT NW composite film (83.8 μW m^–1^ K^–2^) and MXene/PEDOT:PSS composites (155.0 μW m^–1^ K^–2^), which might be because solvent treatment was employed in the earlier work.^27,41^

**Figure 7:**
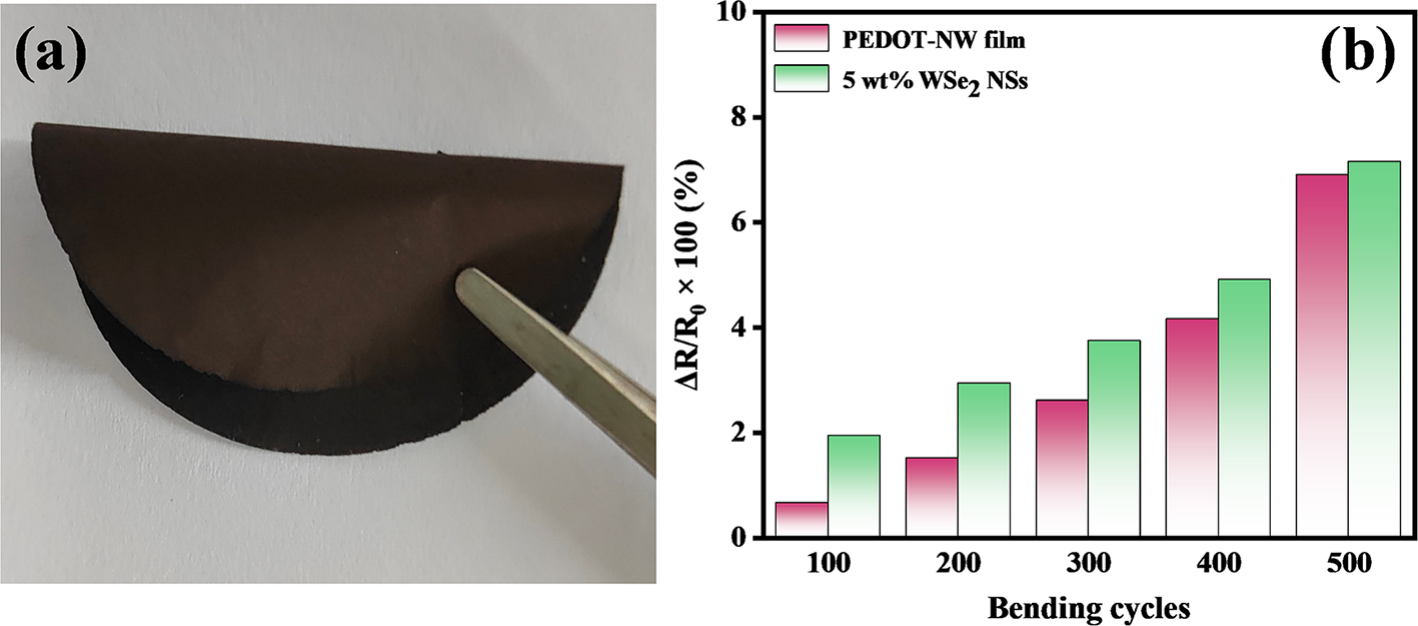
(a) Photo of the vacuum-filtrated freestanding WSe_2_-NS/PEDOT-NW composite film. (b) Resistance change ratios of the PEDOT-NW film and WSe_2_-NS/PEDOT-NW composite film after bending for different times at a radius of 4 mm. The content of WSe_2_ NSs in the WSe_2_-NS/PEDOT-NW composite film was 5 wt %.

Figure 7a shows a photo of the freestanding WSe_2_-NS/PEDOT-NW composite film. Figure 7b shows the resistance change ratios of the WSe_2_-NS/PEDOT-NW composite film after bending for different cycles. For comparation, the resistance change ratio of the PEDOT-NW film was also measured. For 500 bending cycles, the resistance change ratio of the 5 wt % WSe_2_-NS/PEDOT-NW composite film was 7.1%, which was comparable to that of the freestanding PEDOT-NW film (6.9%).

**Figure 8:**
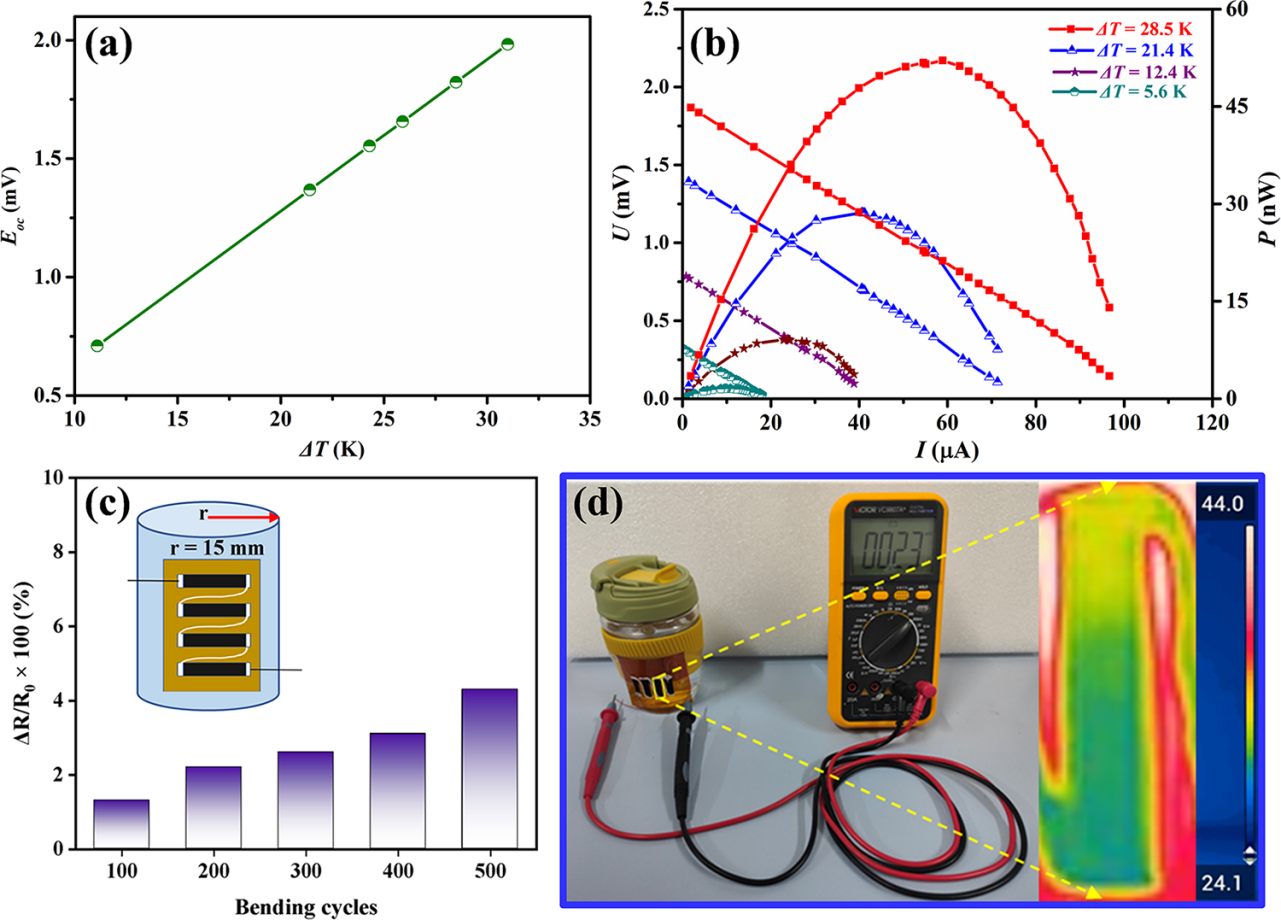
(a) *E*_oc_ as a function of temperature gradient, (b) *U* and *P* vs output current under different temperature gradients of the WSe_2_-NS/PEDOT-NW TE generator. (c) Resistance change ratio of the WSe_2_-NS/PEDOT-NW TE power generator at different bending times. The inset is a schematic diagram of the bending test of the generator. (d) Photo of how *E*_oc_ is generated by the flexible TE generator with hot water (half-filled cup) as the heat source and the corresponding real-time infrared thermal image.

A flexible WSe_2_-NS/PEDOT-NW TE generator was fabricated, and *E*_OC_ generated at different temperature gradients (Δ*T*) is shown in Figure 8a. For Δ*T* of 31 K, the TE generator can generate an *E*_OC_ of 1.98 mV. Figure 8b shows the output of the generator at different Δ*T*. The outputs *U*, *I*, and *P* were obviously increased with Δ*T*, and the TE generator achieved an output power of 52.1 nW (Δ*T* = 28.5 K), which corresponded to the maximal power density (, where *P*_max_ represents the maximal output power, *N* represents the number of TE legs, and *A* represents the cross-sectional area of the TE leg) of ∼0.33 W/m^2^. Figure 8c shows the resistance change ratio of the WSe_2_-NS/P-NW TE generator for different bending times with a bending radius of 15 mm. The TE generator revealed good flexibility since the resistance change ratio was lower than 5% after bending for 500 times. Figure 8d shows a photo of how *E*_oc_ is generated by the flexible TE generator and the corresponding real-time infrared thermal image. It was observed that the flexible TE generator on a drinking glass with hot water could generate an open-circuit voltage of 0.23 mV.

## 4 Conclusions

In summary, PEDOT NWs were synthesized by a modified self-assembled micellar soft-template method. Flexible and freestanding WSe_2_-NS/PEDOT-NW composite films were prepared by a vacuum filtration method. As the mass fractions of WSe_2_ NSs increased from 0 to 20 wt %, the Seebeck coefficient of WSe_2_-NS/PEDOT-NW composite films was increased. A power factor of 44.5 μW m^–1^ K^–2^ was achieved at 300 K when the content of WSe_2_ NSs was 5 wt %, and it was increased to 67.3 μW m^–1^ K^–2^ when the temperature increased to 380 K. The self-supporting WSe_2_-NS/PEDOT-NW composite films were mechanically flexible, and a flexible four-leg thermoelectric power generator was assembled, generating an output power of 52.1 nW at a temperature difference of 28.5 K. This work thus shows that the preparation of inorganic nanosheet/organic nanowire composites is an approach to improve the TE properties of conducting polymers.

## References

[ref1] He J.; Tritt T. M. Advances in thermoelectric materials research: Looking back and moving forward. Science 2017, 357, eaak9997 10.1126/science.aak9997. 28963228

[ref2] Snyder G. J.; Toberer E. S. Complex thermoelectric materials. Nat. Mater. 2008, 7, 105–114. 10.1038/nmat2090. 18219332

[ref3] Freer R.; Powell A. V. Realising the potential of thermoelectric technology: a Roadmap. J. Mater. Chem. C 2020, 8, 441–463. 10.1039/C9TC05710B.

[ref4] Beretta D.; Neophytou N.; Hodges J. M.; Kanatzidis M. G.; Narducci D.; Martin- Gonzalez M.; Beekman M.; Balke B.; Cerretti G.; Tremel W.; Zevalkink A.; Hofmann A. I.; Müller C.; Dörling B.; Campoy-Quiles M.; Caironi M. Thermoelectrics: From history, a window to the future. Mater. Sci. Eng., R 2019, 138, 100501 10.1016/j.mser.2018.09.001.

[ref5] LeBlanc S. Thermoelectric generators: Linking material properties and systems engineering for waste heat recovery applications. Sustainable Mater. Technol. 2014, 1–2, 26–35. 10.1016/j.susmat.2014.11.002.

[ref6] Du Y.; Chen J. G.; Meng Q. F.; Dou Y. C.; Xu J. Y.; Shen S. Z. Thermoelectric materials and devices fabricated by additive manufacturing. Vacuum 2020, 178, 109384 10.1016/j.vacuum.2020.109384.

[ref7] Kim C. S.; Lee G. S.; Choi H.; Kim Y. J.; Yang H. M.; Lim S. H.; Lee S. G.; Cho B. J. Structural design of a flexible thermoelectric power generator for wearable applications. Appl. Energy 2018, 214, 131–138. 10.1016/j.apenergy.2018.01.074.

[ref8] Du Y.; Xu J. Y.; Paul B.; Eklund P. Flexible thermoelectric materials and devices. Appl. Mater. Today 2018, 12, 366–388. 10.1016/j.apmt.2018.07.004.

[ref9] Wang Y.; Yang L.; Shi X. L.; Shi X.; Chen L. D.; Dargusch M. S.; Zou J.; Chen Z. G. Flexible thermoelectric materials and generators: challenges and innovations. Adv. Mater. 2019, 31, 1807916 10.1002/adma.201807916. 31148307

[ref10] Villalva D. R.; Haque M. A.; Nugraha M. I.; Baran D. Enhanced thermoelectric performance and lifetime in acid-doped PEDOT:PSS films via work function modification. ACS Appl. Energy Mater. 2020, 3, 9126–9132. 10.1021/acsaem.0c01511.

[ref11] Bubnova O.; Crispin X. Towards polymer-based organic thermoelectric generators. Energy Environ. Sci. 2012, 5, 9345–9362. 10.1039/c2ee22777k.

[ref12] Bubnova O.; Khan Z. U.; Malti A.; Braun S.; Fahlman M.; Berggren M.; Crispin X. Optimization of the thermoelectric figure of merit in the conducting polymer poly(3,4-ethylenedioxythiophene). Nat. Mater. 2011, 10, 429–433. 10.1038/nmat3012. 21532583

[ref13] Chaudhary N.; Singh A.; Aswal D. K.; Bharti M.; Sharma A.; Tillu A. R.; Roy M.; Singh B. P.; Bahadur J.; Putta V.; Debnath A. K. High energy electron beam induced improved thermoelectric properties of PEDOT:PSS films. Polymer 2020, 202, 122645 10.1016/j.polymer.2020.122645.

[ref14] Ni D.; Song H. J.; Chen Y. X.; Cai K. F. Free-standing highly conducting PEDOT films for flexible thermoelectric generator. Energy 2019, 170, 53–61. 10.1016/j.energy.2018.12.124.

[ref15] Hu X. C.; Chen G. M.; Wang X.; Wang H. F. Tuning thermoelectric performance by nanostructure evolution of a conducting polymer. J. Mater. Chem. A 2015, 3, 20896–20902. 10.1039/C5TA07381B.

[ref16] Han M. G.; Foulger S. H. Facile synthesis of poly(3,4-ethylenedioxythiophene) nanofibers from an aqueous surfactant solution. Small 2006, 2, 1164–1169. 10.1002/smll.200600135. 17193583

[ref17] Zhang J.; Zhang K.; Xu F. J.; Wang S.; Qiu Y. P. Thermoelectric transport in ultrathin poly(3,4-ethylenedioxythiophene) nanowire assembly. Composites, Part B 2018, 136, 234–240. 10.1016/j.compositesb.2017.10.037.

[ref18] Wu M.; Xiao Y. H.; Zeng Y.; Zhou Y. L.; Zeng X. B.; Zhang L. N.; Liao W. G. Synthesis of two-dimensional transition metal dichalcogenides for electronics and optoelectronics. InfoMat 2021, 3, 362–396. 10.1002/inf2.12161.

[ref19] Kim W. Y.; Kim H. J.; Hallam T.; McEvoy N.; Gatensby R.; Nerl H. C.; O’Neill K.; Siris R.; Kim G. T.; Duesberg G. S. Field-dependent electrical and thermal transport in polycrystalline WSe_2_. Adv. Mater. Interfaces 2018, 5, 1701161 10.1002/admi.201701161.

[ref20] Chen K. X.; Luo Z. Y.; Mo D. C.; Lyu S. S. WSe_2_ nanoribbons: new high-performance thermoelectric materials. Phys. Chem. Chem. Phys. 2016, 18, 16337–16344. 10.1039/C6CP02456D. 27254307

[ref21] Jiang F. X.; Xiong J. H.; Zhou W. Q.; Liu C. C.; Wang L. Y.; Zhao F.; Liu H. X.; Xu J. K. Use of organic solvent-assisted exfoliated MoS_2_ for optimizing the thermoelectric performance of flexible PEDOT:PSS thin films. J. Mater. Chem. A 2016, 4, 5265–5273. 10.1039/C6TA00305B.

[ref22] Li X.; Liu C. C.; Wang T. Z.; Wang W. F.; Wang X. D.; Jiang Q. L.; Jiang F. X.; Xu J. K. Preparation of 2D MoSe_2_/PEDOT:PSS composite and its thermoelectric properties. Mater. Res. Exp. 2017, 4, 116410 10.1088/2053-1591/aa99f9.

[ref23] Wang T. Z.; Liu C. C.; Wang X. D.; Li X.; Jiang F. X.; Li C. C.; Hou J.; Xu J. K. Highly enhanced thermoelectric performance of WS_2_ nanosheets upon embedding PEDOT:PSS. J. Polym. Sci., Part B: Polym. Phys. 2017, 55, 997–1004. 10.1002/polb.24349.

[ref24] Chen Y. X.; Shi X. L.; Zheng Z. H.; Li F.; Liu W. D.; Chen W. Y.; Li X. R.; Liang G. X.; Luo J. T.; Fan P.; Chen Z. G. Two-dimensional WSe_2_/SnSe p-n junctions secure ultrahigh thermoelectric performance in n-type Pb/I Co-doped polycrystalline SnSe. Mater. Today Phys. 2021, 16, 100306 10.1016/j.mtphys.2020.100306.

[ref25] Liu W.; Kang J. H.; Sarkar D.; Khatami Y.; Jena D.; Banerjee K. Role of metal contacts in designing high-performance monolayer n-type WSe_2_ field effect transistors. Nano Lett. 2013, 13, 1983–1990. 10.1021/nl304777e. 23527483

[ref26] Kumar S.; Schwingenschlögl U. Thermoelectric response of bulk and monolayer MoSe_2_ and WSe_2_. Chem. Mater. 2015, 27, 1278–1284. 10.1021/cm504244b.

[ref27] Du M. Z.; Chen X. Y.; Zhang K. Origins of enhanced thermoelectric transport in free-standing PEDOT nanowires film modulated with ionic liquid. ACS Appl. Energy Mater. 2021, 4, 4070–4080. 10.1021/acsaem.1c00422.

[ref28] Song H. J.; Cai K. F. Preparation and properties of PEDOT:PSS/Te nanorod composite films for flexible thermoelectric power generator. Energy 2017, 125, 519–525. 10.1016/j.energy.2017.01.037.

[ref29] Meng Q. F.; Jiang Q. L.; Cai K. F.; Chen L. D. Preparation and thermoelectric properties of PEDOT:PSS coated Te nanorod/PEDOT:PSS composite films. Org. Electron. 2019, 64, 79–85. 10.1016/j.orgel.2018.10.010.

[ref30] Aasmundtveit K. E.; Samuelsen E. J.; Pettersson L. A. A.; Inganäs O.; Johansson T.; Feidenhans’l R. Structure of thin films of poly(3,4-ethylenedioxythiophene). Synth. Met. 1999, 101, 561–564. 10.1016/S0379-6779(98)00315-4.

[ref31] Chen B.; Chen Q. L.; Xiao S. H.; Feng J. S.; Zhang X.; Wang T. H. Giant negative thermopower of ionic hydrogel by synergistic coordination and hydration interactions. Sci. Adv. 2021, 7, 7233 10.1126/sciadv.abi7233. PMC8612679 34818039

[ref32] D’Arcy J. M.; El-Kady M. F.; Khine P. P.; Zhang L.; Lee S. H.; Davis N. R.; Liu D. S.; Yeung M. T.; Kim S. Y.; Turner C. L.; Lech A. T.; Hammond P. T.; Kaner R. B. Vapor-phase polymerization of nanofibrillar poly(3,4-ethylenedioxythiophene) for supercapacitors. ACS Nano 2014, 8, 1500–1510. 10.1021/nn405595r. 24490747

[ref33] Ni D.; Song H. J.; Chen Y. X.; Cai K. F. Significantly enhanced thermoelectric performance of flexible PEDOT nanowire film via coating Te nanostructures. J. Materiomics 2020, 6, 364–370. 10.1016/j.jmat.2019.07.001.

[ref34] Wang X. Q.; Chen Y. F.; Zheng B. J.; Qi F.; He J. R.; Li Q.; Li P. J.; Zhang W. L. Graphene-like WSe_2_ nanosheets for efficient and stable hydrogen evolution. J. Alloys Compd. 2017, 691, 698–704. 10.1016/j.jallcom.2016.08.305.

[ref35] Pataniya P. M.; Bhakhar S. A.; Tannarana M.; Zankat C.; Patel V.; Solanki G. K.; Patel K. D.; Jha P. K.; Late D. J. L.; Sumesh C. K. Highly sensitive and flexible pressure sensor based on two-dimensional MoSe_2_ nanosheets for online wrist pulse monitoring. J. Colloid Interface Sci. 2021, 584, 495–504. 10.1016/j.jcis.2020.10.006. 33129159

[ref36] Liu Y. C.; Liu J. F.; Tan X.; Li Y. M.; Liu R.; Lin Y. H.; Nan C. W. High-temperature electrical and thermal transport behaviors in layered structure WSe_2_. J. Am. Ceram. Soc. 2017, 100, 5528–5535. 10.1111/jace.15069.

[ref37] Song H. J. C.; Liu C.; Zhu H. F.; Kong F. F.; Lu B. Y.; Xu J. K.; Wang J. W.; Zhao F. Improved thermoelectric performance of free-standing PEDOT:PSS/Bi_2_Te_3_ films with low thermal conductivity. J. Electron. Mater. 2013, 42, 1268–1274. 10.1007/s11664-013-2587-y.

[ref38] Chetty R.; Bali A.; Naik M. H.; Rogl G.; Rogl P.; Jain M.; Suwas S.; Mallik R. C. Thermoelectric properties of Co substituted synthetic tetrahedrite. Acta Mater. 2015, 100, 266–274. 10.1016/j.actamat.2015.08.040. 25463306

[ref39] Wang X. D.; Meng F. L.; Tang H. T.; Gao Z. M.; Li S.; Jiang F. X.; Xu J. K. An effective dual-solvent treatment for improving the thermoelectric property of PEDOT:PSS with white graphene. J. Mater. Sci. 2017, 52, 9806–9818. 10.1007/s10853-017-1166-7.

[ref40] Guan X.; Ouyang J. Y. Enhancement of the Seebeck coefficient of organic thermoelectric materials via energy filtering of charge carriers. CCS Chem. 2021, 3, 2415–2427. 10.31635/ccschem.021.202101069.

[ref41] Guan X.; Feng W.; Wang X. Z.; Venkatesh R.; Ouyang J. Y. Significant enhancement in the Seebeck coefficient and power factor of p-type poly(3,4-ethylenedioxythiophene):poly(styrenesulfonate) through the incorporation of n-type MXene. ACS Appl. Mater. Interfaces 2020, 12, 13013–13020. 10.1021/acsami.9b21185. 32097550

